# Control of ultrafast hot electron dynamics in epsilon-near-zero conductive oxide thin films

**DOI:** 10.1126/sciadv.adu8850

**Published:** 2025-05-21

**Authors:** Sudip Gurung, Subhajit Bej, Quynh Dang, Ambaresh Sahoo, Aleksei Anopchenko, Zhenhuan Yi, Alexei V. Sokolov, Andrea Marini, Ho Wai Howard Lee

**Affiliations:** ^1^ Department of Physics and Astronomy, University of California, Irvine, CA 92697, USA.; ^2^ The Institute for Quantum Science and Engineering, Texas AM University, College Station, TX 77843, USA.; ^3^ Photonics Laboratory, Tampere University, FI-33720 Tampere, Finland.; ^4^ Beckman Laser Institute and Medical Clinic, University of California, Irvine, CA 92697, USA.; ^5^ Eddleman Quantum Institute, University of California, Irvine, CA 92697, USA.; ^6^ Department of Physical and Chemical Sciences, University of L’Aquila, Via Vetoio, I-67100 L’Aquila, Italy.

## Abstract

The dynamics of nonlinear optical processes in epsilon-near-zero (ENZ) transparent conductive oxides (TCOs) are primarily governed by hot electron relaxation with a sub-picosecond response. However, there is currently a lack of comprehensive understanding of the ultrafast electron dynamics in nonlinear TCO ENZ materials. This study investigates the effects of laser peak power and ENZ mode excitation on hot electron relaxation in TCOs. Our experimental analysis theoretically supported by a hydrodynamic model reveals that increasing laser pulse intensity extends hot electron relaxation time by more than 200%, while ENZ mode excitation increases it by more than 40% in representative TCO ENZ materials. This research demonstrates the controllable modulation of ultrafast ENZ nonlinearity via pulse peak power and ENZ mode field enhancement. These findings provide substantial insights into the potential utilization of ENZ nonlinearity for the development of optical and quantum computing components, including ultrafast optical switches, dynamic pulse shapers, and modulators.

## Teaser

Laser power and mode excitation control hot electron relaxation, enabling ultrafast optical nonlinearity.

## INTRODUCTION

In recent years, there has been growing interest in exploring the optical response of plasmonic transparent conductive oxides (TCOs), which are characterized by low electron densities and a wide energy bandgap. Notably, these materials feature epsilon-near-zero (ENZ) regions where the real part of the electrical permittivity (ε) approaches zero in the near-infrared spectral range (*1*–*3*). ENZ materials exhibit unique phenomena, including ENZ mode excitation in thin TCO films, resulting in strong field confinement and absorption (*1*, *4*–*7*). In addition, TCO ENZ materials offer benefits in the nonlinear optical regime for high harmonic generation (*8*–*10*), efficient ultraviolet light generation (*11*), supercontinuum generation (*8*), and an enhanced optical Kerr effect (*12*–*14*). Their chemical robustness and high optical damage thresholds render them suitable for strong field (extreme) nonlinear light-matter interactions (*15*).

Owing to the ultrafast dynamics of free electrons, TCOs exhibit sub-picosecond nonlinear modulation and relaxation time—the time required for energetic electrons to equilibrate with the lattice (*13*, *14*, *16*)—crucial for ultrafast all-optical applications (*14*, *17*). With a low specific heat capacity, conduction-band electrons in TCOs can be rapidly elevated to high energies under illumination by ultrashort pulsed light with energy below the bandgap (*12*, *18*–*20*). These nonthermal electrons swiftly equilibrate through electron-electron collisions (EECs), forming a hot electron distribution. Subsequently, this distribution relaxes via electron-phonon scattering, where the hot electrons exist in an out-of-equilibrium (OOE) state with the lattice. This important alteration markedly modifies the optical properties of ENZ materials (*12*, *15*). Typically, the two-temperature model (TTM) is used to study hot electron dynamics in TCOs, because of its simplicity and intuitive representation of the process. TTM assumes instantaneous thermalization of the nonthermal electron distribution into a hot Fermi distribution due to EECs, followed by cooling through electron-phonon collisions (EPCs) with the phonon subsystem. However, for short laser pulses (<<100 fs), the assumption of “instantaneous thermalization” is generally invalid, as it neglects the critical role of EECs (*21*, *22*).

Although understanding hot electron dynamics in metals requires insight into both EECs and EPCs (*22*), recent studies on TCOs confirm that the hot electron relaxation time primarily depends on EPCs (*20*, *23*). TCOs such as indium tin oxide (ITO) and aluminum-doped zinc oxide (*24*) exhibit notably higher EPC rates compared to metals, particularly in their ENZ regions, leading to a more rapid decrease in electron temperature, and subsequently a faster optical response (*20*). Another study also reveals that the TTM is generally sufficient for pulse durations longer than the thermalization time, but an extended TTM is necessary for shorter pulses to account for nonthermal electrons (*23*). Unlike the traditional TTM, the extended TTM accounts for the finite EEC time, allowing the electron subsystem to be nonthermal at femtosecond scale transients. This study also highlights the impact of illumination intensity on hot electron relaxation. High illumination intensities can lead to a substantial increase in the real part of the permittivity, which causes the ENZ resonance to shift away from the incoming frequencies. This, in turn, increases the electron temperature and changes the dynamics of the electron distribution, making the relaxation time faster as the illumination intensity increases (*23*). In a recent study, Bykov *et al.* (*24*) used the TTM model to describe the temporal response of photoexcited hot electrons and observed ultrafast optical modulation in thin ITO films, attributing it to changes in plasma frequency and the Drude damping constant at increased electron temperatures. Wang *et al.* (*18*, *19*) adopted another approach to investigate the relaxation dynamics in ITO by incorporating multiple scattering mechanisms and examining their dependence on electron temperature. Extending the Drude model to the nonlinear regime, they found that the temperature-dependent damping rate was primarily influenced by ionized impurities and acoustic phonon scattering, increasing at higher electron temperatures. This contrasts with previous studies (*20*, *25*, *26*) and highlights the need for further research on ultrafast ENZ nonlinearity.

Existing research on ENZ nonlinear materials does not provide comprehensive insight into the ultrafast characteristics of ENZ nonlinearities, their dependence on ENZ mode excitation, and laser pulse intensity. Therefore, it is important to rigorously examine the temporal dynamics of hot electrons to fully understand the ultrafast optical response of ENZ materials. This work aims to detail the relationship between relaxation time and the application of high laser pulse intensity, as well as the electric field intensity enhancement (FIE) due to ENZ mode excitation. We use an advanced hydrodynamic model (HDM) to describe the ultrafast hot electron dynamics resulting from nonlinear electron collisions, accounting nonperturbatively for EECs and EPCs (*27*). This is accomplished by the nonperturbative evaluation of the Boltzmann collision integral in the Landau weak coupling assumption and the solution of Boltzmann equation by the method of moments, leading to a modified HDM where EPCs produce intensity-dependent dephasing, heating, and relaxation rates (*27*). In the considered HDM, EECs are accounted to produce an OOE electron temperature that can deviate substantially from the lattice temperature. The approximation adopted in the HDM derivation is that EECs produce an instantaneous electron thermalization as they conserve the total energy. Moreover, because EECs conserve the total momentum and particle number, they do not explicitly enter the spatiotemporal PDEs governing the zeroth- and first-order distribution moments (electron density and mean velocity, respectively) even if a finite collision time is accounted (*28*). Our model quantitatively predicts the nonlinear relationship between hot electron relaxation time and the excitation of intense laser pulses and ENZ mode, demonstrating controllable modulation of ultrafast ENZ nonlinearity. This is confirmed by degenerate pump-probe measurements on AZO and ITO thin films with thicknesses of 205 and 306 nm and ENZ wavelengths of 1480 and 1190 nm, respectively. Further details on the optical properties of the ENZ thin films are provided in section S1.

## RESULTS

### Theoretical model for ultrafast hot electron dynamics

The ultrafast dynamics of electrons in plasmonic materials typically involve complex relaxation processes that include electron heating and subsequent energy transfer from hot electrons to the lattice via electron-phonon scattering. We use the HDM with a semiclassical approach, treating the conduction band electrons as a free-electron gas immersed in a homogeneous background of ions at ambient temperature (*29*). Starting from the time-dependent Boltzmann equation for the nonequilibrium electron distribution function accounting for EECs and EPCs through the full nonlinear collisional integrals, we derive a Fokker-Planck-Landau equation specialized for plasmonic materials under the assumption of weak coupling (*29*). The Fokker-Planck-Landau approach is well established in plasma physics and OOE statistical mechanics for modeling fast electron relaxation in Tokamaks (*30*).

**Fig. 1.**
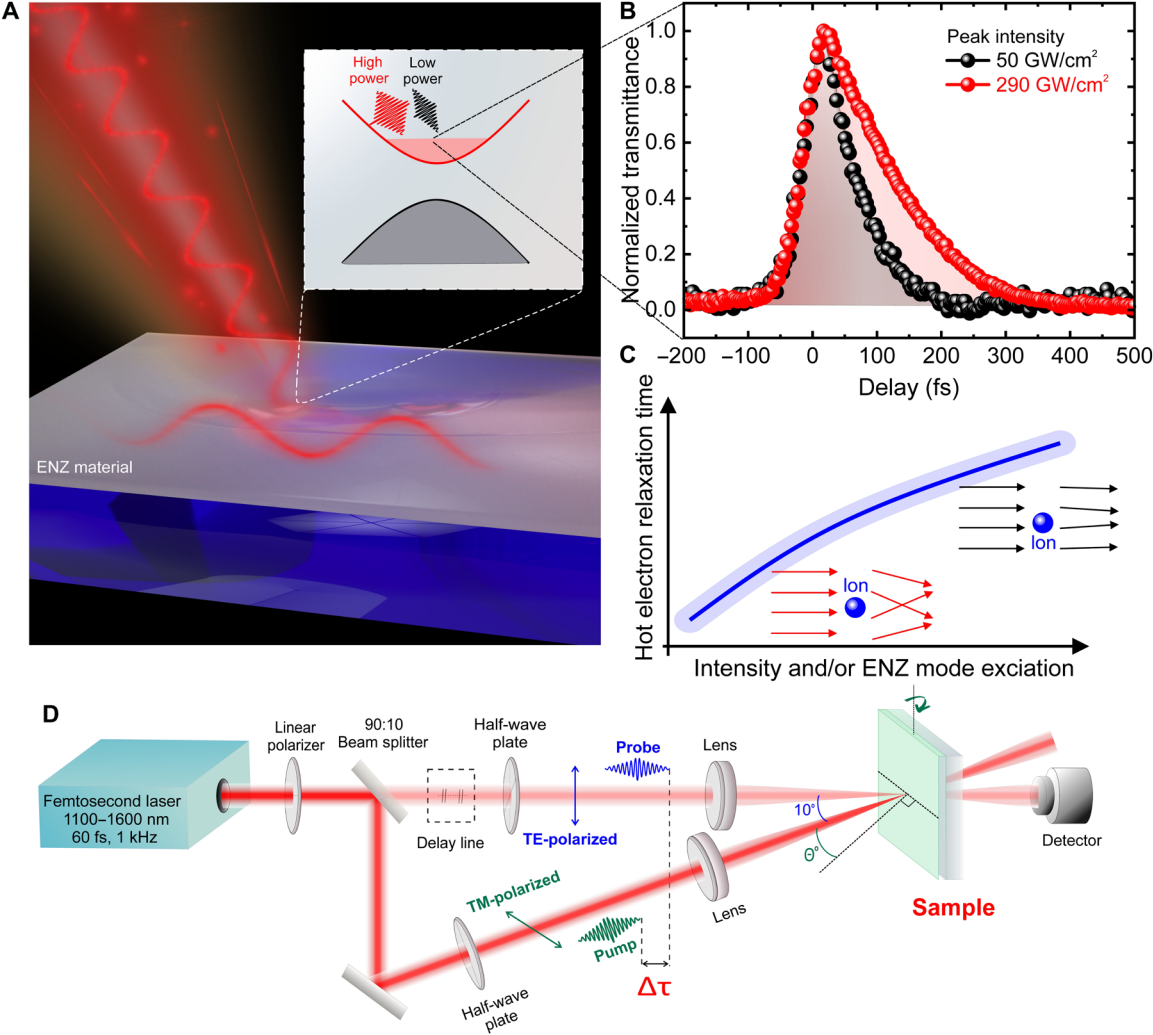
Overview schematic demonstration of the hot electron excitation and experimental setup. (**A**) Schematic of the ultrafast pulse interaction with ENZ material. (**B**) Normalized experimental transmittance Δ*T*/Δ*T*_max_ versus delay time at 50 GW/cm^2^ (black circles) and 290 GW/cm^2^ (red circles) for AZO films. (**C**) Cartoon representation of hot electron relaxation time dependence on pump laser peak intensity (*I*_peak_) and/or ENZ mode excitation (θ,λ). The black arrows represent electrons with higher temperatures and kinetic energies, while the red arrows indicate electrons with lower temperatures and kinetic energies. Upon interaction with ions (depicted as blue spheres), electrons with lower kinetic energy experience greater deflection compared to those with higher kinetic energy. (**D**) Schematic of ultrafast degenerate pump-probe measurement setup.

By analytically calculating the Rosenbluth potentials, we obtain a novel set of hydrodynamical equations accounting for both local and nonlocal electron dynamics. Moreover, optical energy transfer to the electron plasma is highly reduced for OOE electrons with average velocities higher than thermal velocity, leading to damping quenching (*29*). Intuitively, damping quenching for highly energetic electrons can be understood as reduced coulomb interaction between the electrons and ions owing to the shorter interaction time. As electrons gain more energy, they experience less deflection during collisions, resulting in a diminished damping effect. The lowered deflection of highly energetic electrons leads to a lower probability of EPCs, resulting in increased hot electron relaxation time ($\tau_{r}$) (see Fig. 1, A and C). Similar to damping quenching, thermalization of the hot electron gas with the lattice is also quenched for large intensity excitation. At large field intensity excitation, elevated hot electron temperatures exhibit increased electron thermal velocities, reducing the interaction time between electrons and ions and consequently quenching the thermalization rate. The perturbative solution of the HDM provides an analytical expression for the hot electron relaxation rate ($\gamma_{r}$)

$$\gamma_{r}\approx \gamma_{\text{th}}-\frac{\gamma_{\text{th}}\left(2\gamma -\gamma_{\text{th}}\right)e^{2}f\left(\theta ,\omega \right)}{12\gamma k_{B}T_{0}m\varepsilon_{0}c\left(\omega^{2}+\gamma^{2}\right)}I_{0}$$ (1)

where γ is the linear dephasing rate (Drude damping), $k_{B}$ is the Boltzmann constant, $T_{0}$ is the lattice temperature, m is the electron effective mass, ω is the carrier angular frequency of impinging radiation, ${\gamma_{\text{th}}}^{-1}$ is the linear thermalization time, and e and c are the charge of an electron and the speed of light, respectively. Equation 1 illustrates the dependence of the hot electron relaxation rate on the intensity of the impinging radiation ($I_{0}$) and on the FIE factor f(θ,ω) caused by the excitation of ENZ mode. Thus, the electron relaxation dynamics slow down as the FIE increases due to ENZ mode excitation (e.g., by varying the excitation wavelength or angle of incidence). It should be noted that the FIE can also be increased by reducing the loss or thickness of the ENZ film via fabrication (*5*, *31*). More information on the FIE of the ENZ thin films in this study can be found in section S2.

### Experimental demonstration of intensity-dependent hot electron relaxation time

**Fig. 2.**
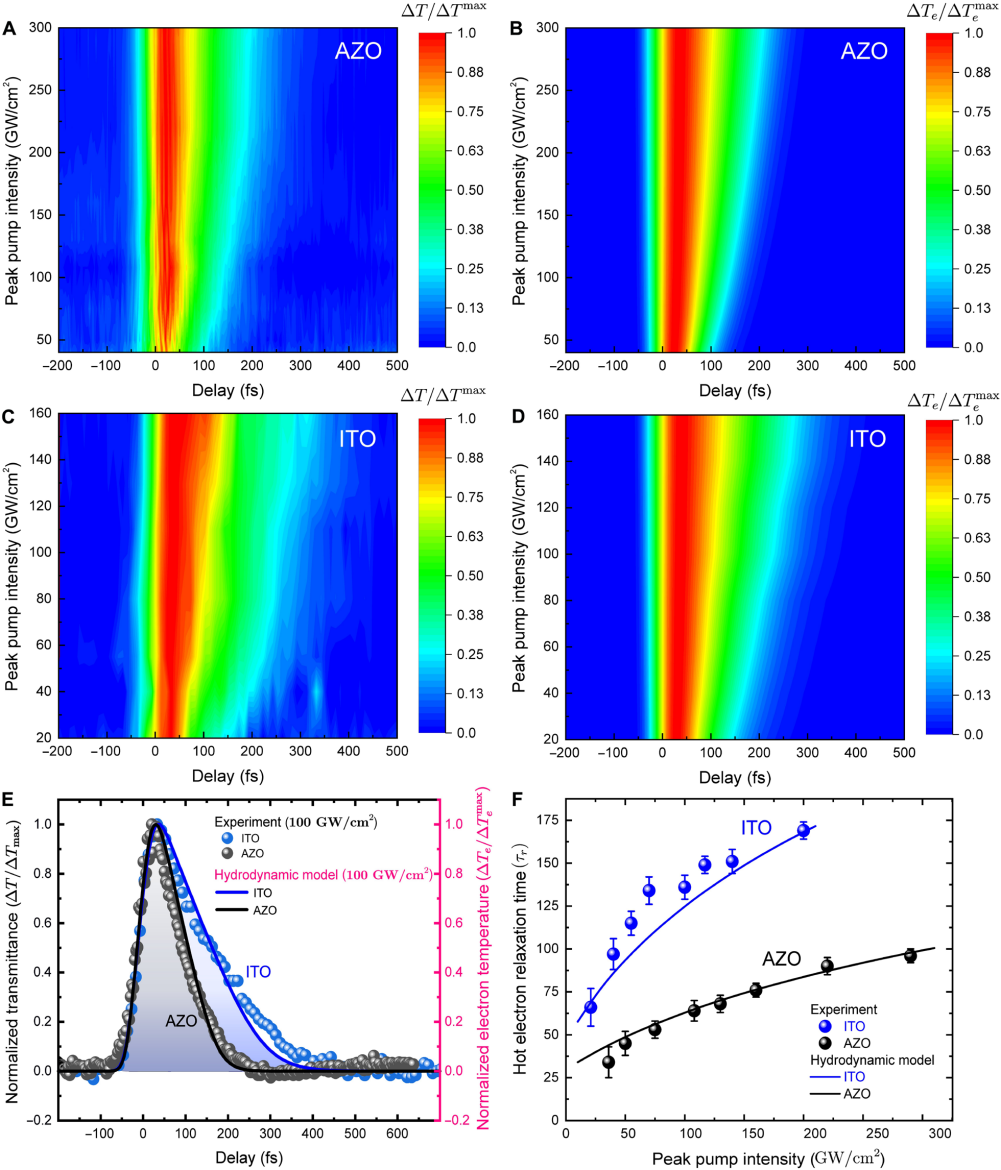
Intensity-dependent hot electron relaxation in AZO and ITO samples. (**A** and **C**) Normalized experimental transmittance (Δ*T*/Δ*T*_max_) and (**B** and **D**) normalized electron temperature ($∆T_{e}\;/\;∆T_{e}^{\text{max}}$) versus delay at different peak pump intensities for AZO and ITO, respectively. (**E**) Normalized experimental transmittance (Δ*T*/Δ*T*_max_) and normalized electron temperature ($∆T_{e}\;/\;∆T_{e}^{\text{max}}$) versus delay time at 100 GW/cm^2^ for AZO and ITO films [circles: experimental data for normalized transmittance (left *y* axis, black), solid lines: HDM prediction for the normalized electron temperature (right *y* axis, pink)]. (**F**) Comparison of the magnitude of hot electron relaxation time $\;(\tau_{r})$ at different peak pump intensities for AZO and ITO films. Circles are obtained by fitting the experimental data using the TTM, and lines are theoretical HDM calculations. For the intensity-dependent measurements, the pump and probe wavelengths are fixed at the respective ENZ wavelengths—1480 nm for AZO and 1190 nm for ITO. The pump beam in these measurements is incident normally on the sample.

We experimentally verify the controllable electron relaxation dynamics by performing pump-probe measurements on AZO and ITO thin films at normal incidence and their respective ENZ wavelengths (Fig. 1D). The experimental normalized transmittances for peak pump intensity of 50 and 290 GW/cm^2^ for the AZO sample are shown in Fig. 1B. A strong dependence between the relaxation time and the pump intensity is observed. We further measure for both AZO and ITO samples with different peak pump intensities, and the corresponding experimental normalized transmittance and the theoretical HDM normalized electron temperature are depicted in Fig. 2 (A and C, and B and D, respectively). We observed a good agreement between the experimental and theoretical results. The relaxation time for the normalized transmittance increases with peak pump intensity (Fig. 2, A and C), which is consistent with the simulated normalized electron temperature versus intensity using the HDM (Fig. 2, B and D). To determine the hot electron relaxation time ($\tau_{r}$) from the experiment, the normalized transmittance from the pump-probe experiment was fitted using the same dependence as the normalized electron temperature in the TTM, as the change in electron temperature governs the change in transmittance (*20*) (see eq. S23 in section S5). The direct correspondence between the normalized transmittance and the normalized change in electron temperature has been confirmed, validating our approach (as shown in section S5; fig. S4, B and C). A comparison of the normalized transmittance experimental data and the normalized electron temperature calculated using the HDM for AZO and ITO at 100 GW/cm^2^ is shown in Fig. 2E. The time evolution of the electron temperature is plotted using the HDM by numerically integrating the HDM with a fourth-order Runge-Kutta algorithm (details on the HDM and TTM can be found in sections S4 and S5). Note that the thermalization time ($\tau_{\text{th}}$) for calculating the hot electron relaxation time is estimated from the intensity-dependent pump-probe experiment (see Fig. 2F) by extrapolating the relaxation time to very low intensity. The retrieved $\tau_{\text{th}}$ is then used as a fixed parameter in the HDM with $\tau_{\text{th}}$ values of 21 fs for AZO and 42 fs for ITO (see section S1 and table S1).

As shown in Fig. 2, the relaxation time increases with pump intensity, nearly three times for both AZO (31.4 ± 7 fs at ~20 GW/cm^2^ to 89.6 ± 3 fs at ~200 GW/cm^2^) and ITO (62.6 ± 8 fs at ~20 GW/cm^2^ to 169.5 ± 3 fs at ~200 GW/cm^2^). It is evident from Fig. 2F that the temporal response of AZO is nearly twice as fast as that of ITO, which can be attributed to the shorter linear thermalization time $\tau_{\text{th}}$ and the weaker ENZ mode excitation, represented by f(θ,ω) (see sections S1 and S2, table S1, and fig. S2). The sublinear dependence of relaxation time on laser pulse intensity (Fig. 2F) is due to higher-order contributions to the relaxation rate, the nonlinear decrease of the dephasing rate as intensity increases, and the saturation of thermalization time as electron temperature and thermal velocity rise.

### Control of hot electron dynamics via ENZ mode excitation

**Fig. 3.**
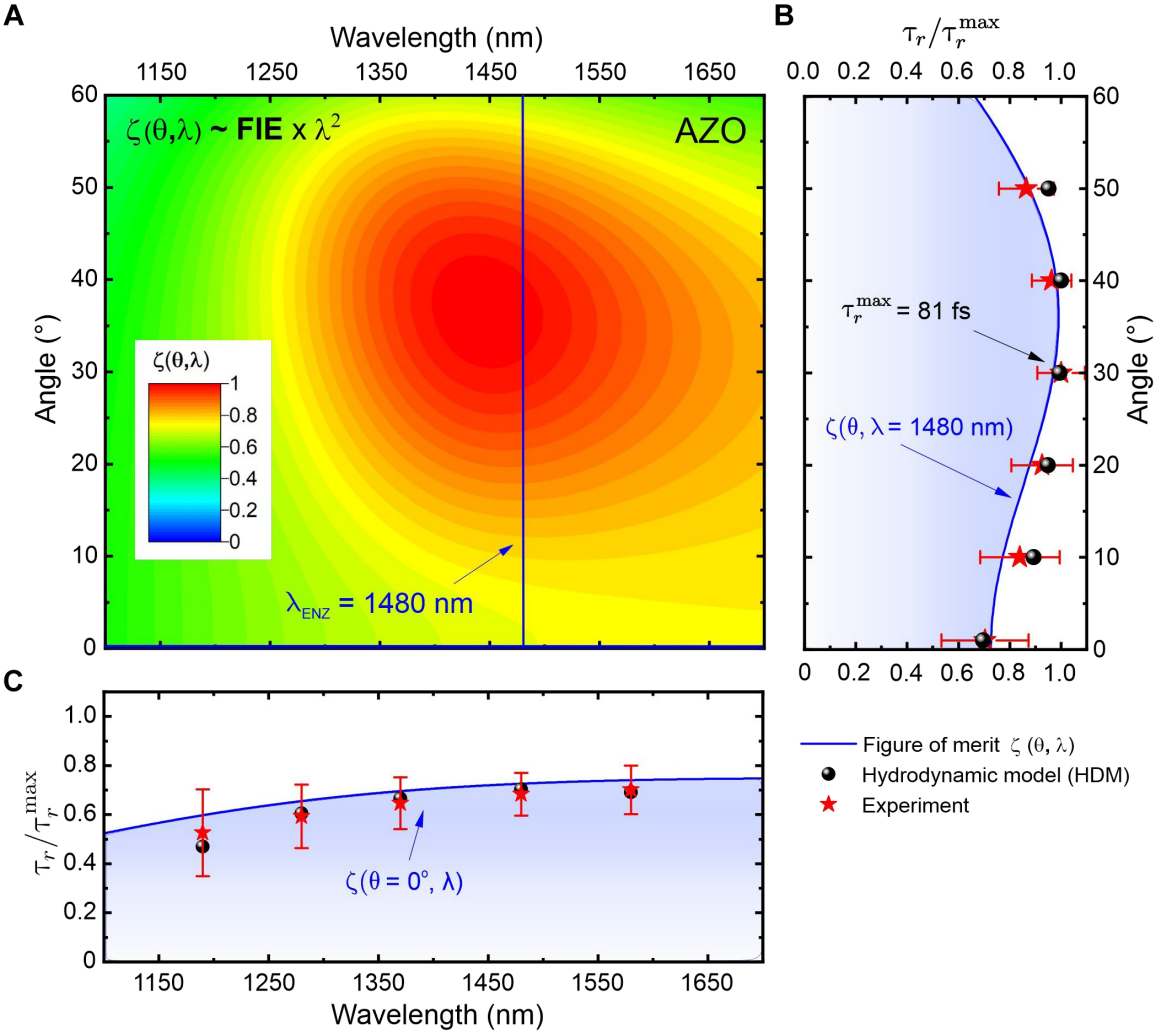
Mode dependence of the relaxation time in the AZO sample. (**A**) Contour map of the figure of merit ζ(θ,λ). The horizontal and vertical blue lines show the coordinates of the contour map profiles/projections on the left and bottom. (**B**) Angular and (**C**) wavelength dependence of the relaxation time at 100 GW/cm^2^. Circles are HDM calculations, stars are the values obtained by fitting experimental data using TMM, and the solid lines are obtained analytically with $\zeta \left(\theta ,\lambda \right)=f\left(\theta ,\lambda \right)\times \lambda^{2}$.

**Fig. 4.**
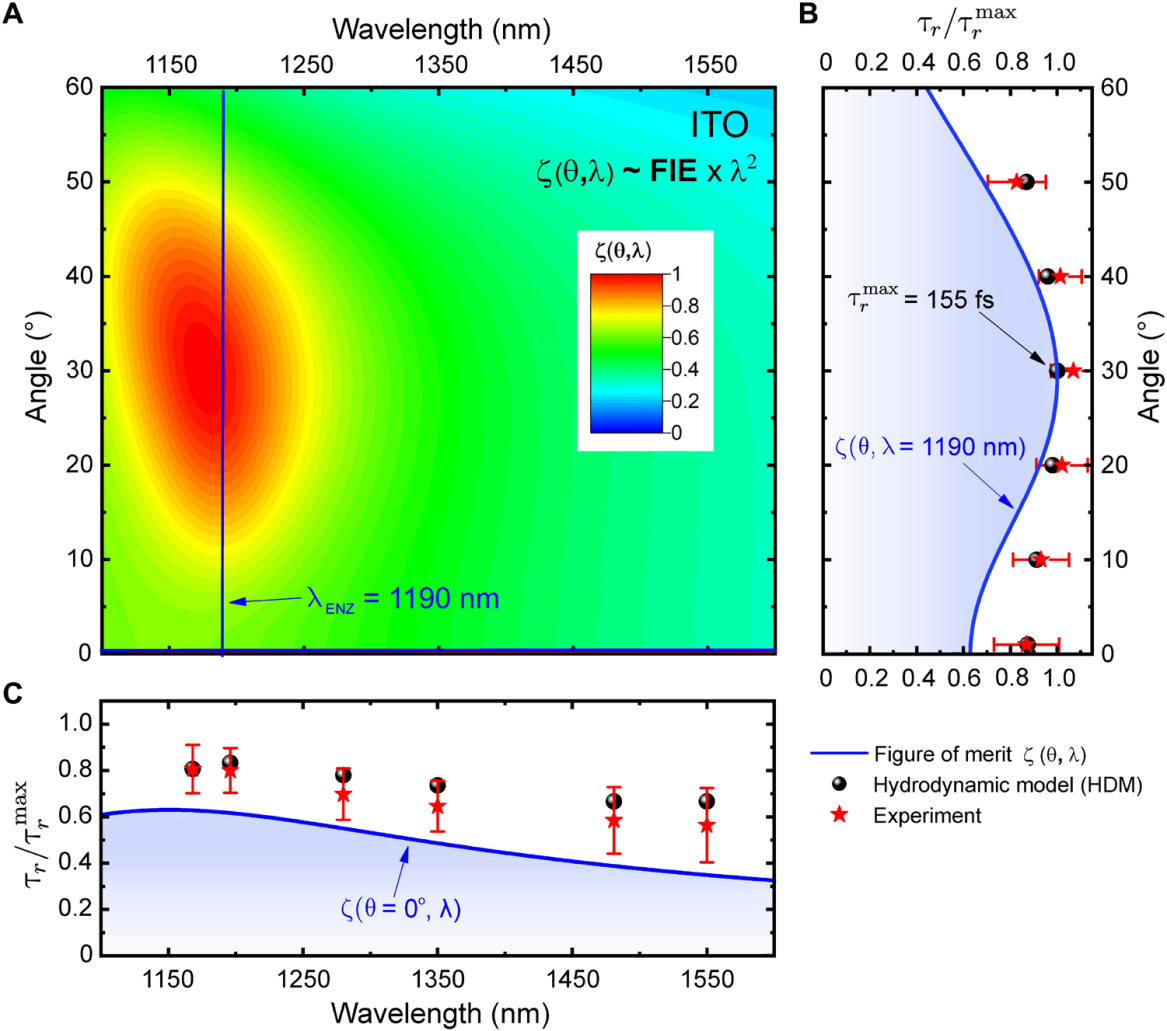
Mode dependence of the relaxation time in the ITO sample. (**A**) Contour map of the figure of merit ζ(θ,λ). The horizontal and vertical blue lines show the coordinates of the contour map profiles/projections on the left and bottom. (**B**) Angular and (**C**) wavelength dependence of the relaxation time at 100 GW/cm^2^. Circles are HDM calculations, stars are the values obtained by fitting experimental data using TMM, and the solid lines are obtained analytically with $\zeta \left(\theta ,\lambda \right)=f\left(\theta ,\lambda \right)\times \lambda^{2}$.

We further investigate how the hot electron relaxation time depends on the ENZ mode, given that the field intensity within the ENZ medium is influenced by ENZ mode excitation. The FIE factor of the ENZ film f(θ,ω) varies substantially with the angle of incidence. According to Eq. 1, the relaxation time exhibits a linear relationship with both f(θ,ω) and the square of the wavelength. Hence, for a fixed intensity, the angular and wavelength dependence of the relaxation time can be predicted using a normalized slowdown measure $\zeta \left(\theta ,\lambda \right)=f\left(\theta ,\lambda \right)\times \lambda^{2}$, which is the product of f(θ,ω) and the square of the wavelength of excitation. Figures 3 and 4 show the theoretically predicted and experimentally obtained hot electron relaxation time dependence on laser pulse wavelength and the angle of incidence of the pump beam for AZO and ITO thin films, respectively. The hot electron relaxation time is normalized to its maximum value, which is 81 fs for AZO and 155 fs for ITO at a 30° incident angle and their respective ENZ wavelengths. As mentioned above, AZO’s hot electron relaxation time is about half that of ITO at a given intensity for various angles of incidence. This difference can be attributed to the shorter linear thermalization time ($\tau_{\text{th}}$) and the weaker ENZ mode excitation, i.e., f(θ,ω) in the AZO sample (fig. S2). It should be noted that the FIE factor f(θ,ω) is loss dependent, i.e., larger for ENZ materials with lower loss ($\varepsilon_{\text{ENZ}}^{\prime \prime }$) (*31*). Hence, the change in f(θ,ω) and the hot electron relaxation time between 20° and 50° of incidence is more pronounced for the ITO 306-nm sample, which has lower loss at the ENZ wavelength, compared to the AZO 205-nm sample [see section S2 for details on f(θ,ω) for both samples]. Furthermore, our excitation geometry leads to radiative ENZ mode excitation, resulting in a weaker angular dependence of f(θ,ω) within the ENZ medium. This results in a less noticeable dependence of the hot electron relaxation time on the incident angles (Figs. 3B and 4B). However, f(θ,ω) can be further enhanced by the excitation of Berreman-type mode (*4*) or propagating ENZ mode, which would substantially increase the hot electron relaxation time.

The angular dependence of the normalized figure of merit ζ(θ,λ) shows a more pronounced peak in the ITO sample due to stronger mode excitation from low loss (see Fig. 4B). For a given wavelength, ζ(θ,λ) exhibits a similar angular dependence as f(θ,ω) [see fig. S2 for the mode-dependent f(θ,ω)]. The hot electron relaxation time peaks around 35° for the AZO sample (Fig. 3B) and near a 30° angle of incidence for the ITO sample (Fig. 4B). At normal incidence, the electron relaxation time ($\tau_{r}$) shows only a minor dependence on wavelength with an unpronounced maximum near the ENZ wavelength and values approximately 20 to 40% lower than those at the peak of ζ(θ,λ). It should be noted that the slight discrepancy between ζ(θ, λ) and both the experimental and HDM results arises from ζ(θ,λ) being a first-order approximation of the relaxation time. Nonetheless, the experimental and HDM calculations are in close agreement. The dependence of hot electron relaxation time on the angle of incidence and wavelength observed in both AZO and ITO indicates that this behavior is typical of all ENZ TCO materials.

## DISCUSSION

By nonperturbatively accounting for EECs and EPCs, the HDM accurately captures nonlinear damping (or dephasing), heating, and relaxation rates. This contrasts with the TTM and extended TTM approaches, where the electron relaxation is typically linear and such effects are often introduced heuristically. In the considered HDM, these dependencies are derived directly from the Boltzmann equation extending previous HDM versions obtained by solving the Boltzmann equation through the method of moments in the relaxation time approximation (*32*), providing the evolution of electron density, mean velocity, and temperature. In the relaxation time approximation, the Boltzmann collisional integral is linearized and is approximated to –γ(*f − f*_0_), where γ is the Drude damping rate, and *f* and *f*_0_ are the OOE and equilibrium plasma distribution functions, respectively. However, the relaxation time approximation completely neglects the nonlinearity produced by collisions. In the considered HDM, we adopt the Landau weak-coupling approach (*28*), where large-deflection collisions are neglected but nonlinearity is maintained. By solving the Boltzmann equation though the method of moments, we achieve the reported HDM, characterized by nonlinear current damping and temperature relaxation rates, playing a leading role in the observed experimental results. We emphasize that, at low fluence, such rates converge to the linear current damping rate and temperature relaxation (*29*), thus reproducing previously adopted HDMs. While, in principle, the HDM could account also for nonlocality arising from the boundaries, the interplay of nonlinear collisions and nonlocality is nontrivial to model as it requires the solution of coupled Maxwell-HDM equations by finite element/difference numerical simulations. While this is feasible in principle, it requires extending currently adopted methods (*33*) to account also for nonlinear collisions. However, because we are considering thin TCO films with thickness > 100 nm in our work, we can neglect the nonlocality. The HDM is applicable to both metals and TCO semiconductors, although macroscopic quantities such as dephasing rate, relaxation time, plasma frequency, and effective mass may vary between them. TCOs exhibit notably shorter relaxation times compared to metals. Specifically, the relaxation times in AZO and ITO are more than 20 times shorter than those in gold. Within the TTM framework, this difference is attributed to TCOs’ lower electron heat capacity $\left(C_{e}\right)$ and higher electron-phonon coupling constant $\left(g_{e-p}\right)$ (*20*).

The differences between AZO and ITO depend on material-specific intrinsic parameters such as plasma frequency, linear dephasing rate (Drude damping), linear relaxation rate, and effective mass (see table S1). Detailed relaxation times for AZO and ITO from both past and current studies are listed in table S2. However, direct comparisons between studies are challenging due to variations in experimental conditions, such as laser repetition rate, pulse width, and wavelengths. Both AZO and ITO show a pronounced increase in relaxation time with higher excitation intensity. For AZO, the relaxation time rises from 31.4 ± 7 fs to 89.6 ± 3 fs as intensity increases from 20 to 200 GW/cm^2^. For ITO, it increases from 62.6 ± 8 fs to 169.5 ± 3 fs over the same intensity range. These results are consistent with observations by Guo *et al.* and Diroll *et al. (*26*, *34*)* who found that relaxation time increases with light intensity in their pump-probe experiments on ENZ colloidal nanocrystals.

The slowdown in hot electron relaxation dynamics due to ENZ mode excitation is linked to a decrease in the EPC rate predicted by the HDM. ENZ mode excitation induces FIE within the TCO film, affecting the hot electron relaxation time (more than 24 fs for AZO and 29 fs for ITO). The comparison of relaxation dynamics in AZO and ITO thin films shows that the electron relaxation time in ITO is twice as long as in AZO at a given intensity, likely due to ITO’s longer linear thermalization time ($\tau_{\text{th}}$) and stronger FIE [f(θ,ω)].

The ENZ mode is supported only by thin films of ENZ materials, where the electric field is strongly enhanced. This makes it important to discuss the thickness dependence of electron relaxation time according to the HDM and determine the valid thickness range for this model. The thickness dependence appears only in the FIE factor f(θ,ω) (see Eq. 1), and the model is independent of thickness and remains valid for films as thin as 10 nm. Below this thickness, finite size effects become dominant. In addition, there is an implicit thickness dependency due to our averaging of the field within the film and the assumption of uniform temperature, which is accurate only for films much thinner than the wavelength. While ITO and AZO can leverage radiative ENZ modes to achieve scaled temporal responses in the near-infrared, it is possible to extend this control from the near- to the mid-infrared by adjusting the material type, composition, and electron density. By selecting materials with suitable optical properties—such as yttrium-doped cadmium oxide (CdO)—it becomes feasible to cover an even wider spectral range, spanning from the mid- to the far-infrared (*35*). In particular, by tuning the electron mobility and carrier density in doped CdO, the spectral position of the ENZ modes can be shifted, enabling tailored ultrafast optical responses over a broad range of wavelengths.

However, ENZ behavior spans a broad spectral range—from UV to FIR—depending on the material system, with nonlinear mechanisms varying across different classes. In TCOs, as studied in our work, nonlinearity arises from hot electrons, with relaxation dynamics governing the ultrafast response. In contrast, polymers and polar materials exhibit ENZ behavior through distinct mechanisms, such as π-bond electron interactions or phonon-driven effects, leading to different relaxation timescales. While TCOs allow direct control of hot electron relaxation, other ENZ materials require alternative strategies for engineering nonlinear dynamics, highlighting the need for further exploration of material-dependent mechanisms.

The measured relaxation times in AZO and ITO are more than 20 times shorter than those reported for gold. In materials where nonlinear optical responses are governed by hot electrons, relaxation time depends on electron concentration, mobility, and thermalization time. Higher carrier concentrations accelerate thermalization but reduce mobility and slowing relaxation. TCOs, with lower carrier concentrations but higher mobility than metals, exhibit faster relaxation under ultrashort laser pulses (<100 fs). Some materials relax even faster, including highly doped semiconductors (e.g., CdO) with tunable carrier densities (*36*), graphene and two-dimensional materials with few-femtosecond dynamics (*37*), and plasmonic nanostructures with strong carrier confinement (*38*). Studying materials with even shorter relaxation times requires advanced laser systems with shorter pulses and higher temporal resolution.

In this work, we compared dynamic transmission changes from pump-probe experiments with the HDM’s predictions of electron relaxation dynamics, focusing on hot electron relaxation time as a function of intensity, angle, and wavelength. Because the model uses a Landau weak-coupling approach, neglecting EECs and EPCs with large deflection angles, it simplifies the collision integral but potentially overlooking strong-field interaction dynamics. Moreover, we emphasize again that the considered calculations have been carried out in the local limit, neglecting HDM nonlocal terms, electron spill out, and the effects of thin field boundaries. Despite this, the HDM’s predicted electron dynamics align well with experimental data. Future work will test its predictions for absorption saturation at high intensities in TCO thin films.

We have demonstrated the ability to control hot electron relaxation dynamics in thin films of TCO ENZ materials using intense femtosecond laser pulses. We observed a noteworthy extension in hot electron relaxation time with increased laser pulse intensity. Building on the development of the HDM, we introduced a figure of merit, ζ(θ,λ), based on the linear optical properties of ENZ thin films. This figure of merit relates the hot electron relaxation time to the angle of incidence and wavelength of light, accounting for the ENZ mode excitation effects. Our findings offer unrevealed insights into ultrafast electron dynamics in ENZ materials. The ability to control hot electron relaxation will be critical for advancing all-optical device components such as ultrafast switches, all-optical transistors, and logic gates, and will also facilitate exploration of various fundamental topics in the context of ENZ nonlinear optics and photonics.

## MATERIALS AND METHODS

### Experiment design: Degenerate pump-probe experiment

To study the temporal dynamics of hot electrons in the transparent conducting ENZ thin film, we measure the change in transmission of the samples in time (in femtoseconds) using a degenerate pump-probe measurement setup. In a degenerate pump-probe measurement, an intense pump pulse and a weak probe pulse, which are generated from a single laser beam, are overlapped in space and time on the sample. The change in probe transmittance is measured based on the time delay between the pulses.

The experiment setup consists of a femtosecond laser Coherent Ti:Sapphire Laser operating at a 1-kHz repetition rate. An external optical parametric amplification (OPA) enables wavelength tuning across a broad spectral range from 1100 to 1600 nm. The laser output is linearly polarized and routed to a degenerate pump-probe setup. A 90:10 beam splitter divides the laser output, creating an intense pump (90% power) and a weak probe beam (10% power). The probe beam propagates through a delay line with a 1-μm (3.3-fs) step size, ensuring precise temporal overlap between the pump and probe pulses within the thin film sample. Pump and probe beams are focused on the sample using convex lenses (focal lengths of 200 and 150 mm, respectively). At the focal point, the pump beam’s radius is 1.33 times that of the probe beam. The sample mount allows rotational positioning relative to the beams. The pump beam is TM-polarized to engage the ENZ mode at an oblique incidence, while the probe beam is TE polarized. The transmission of TE-polarized pulse is independent of incident angles when the pump beam is absent. Comprehensive details of all optical components are provided in table S3.

The pulse duration of femtosecond laser pulses varies with the pulse peak wavelength. Shorter wavelengths necessitate broader bandwidths for temporal confinement, whereas longer wavelengths result in narrower bandwidths and increased pulse durations. A detailed characterization of the pulse duration is shown in fig. S3.

### Sample fabrication

AZO nanolayers were grown on 500-μm–thick fused silica substrates using an ALD reactor (Arradiance GEMSTAR-8). The substrates were cleaned with acetone, isopropanol, and deionized (DI) water, and dried with nitrogen gas. Diethylzinc (*1*, *39*) and DI water were used as precursors, with pulse durations of 30 and 21 ms. Nitrogen gas purging was set at 20 standard cubic centimeters per minute (sccm) for 40 s. For Al-doping, trimethylaluminum (TMA) and DI water were alternately pulsed. AZO films were formed by repeating ZnO and Al cycles, with thickness controlled by macrocycle numbers. ITO was deposited using RF magnetron sputtering (K-lab). The ITO target (90:10 In_2_O_3_) was 120 mm from the substrate holder, with RF power at 50 W and base pressure at 1 × 10^−5^ mbar. Argon flow was 60 sccm, and oxygen flow was adjusted to 5 × 10^−3^ mbar. Substrate temperature was 500°C, and deposition rate was 20 nm/min, achieving 306-nm-thick filmin 900 s.

### FIE [f(θ,ω)] and normalized slowdown measure [ζ(θ,λ)] calculation

Complex refractive indices of ENZ thin films obtained by spectroscopic ellipsometry were used in the numerical simulations. f(θ,ω)can be calculated from the absorptance using the following equation, $f\left(\theta ,\omega \right)={\left|\frac{E}{E_{i}}\right|}^{2}=\frac{A\left(\theta ,\lambda \right)\cdot n_{i}\cdot \text{cos}\left(\theta \right)}{\varepsilon^{\prime \prime }}\frac{\lambda }{2\pi t}$ (*1*, *31*). Here, E is the average field inside the AZO nanolayer, $E_{i}$ is the incident field, $n_{i}$ is the refractive index of the incident medium, θ is the incident angle, $\varepsilon^{\prime \prime }$ is the imaginary permittivity, and A(θ,λ) is the absorptance. The absorptance was calculated using a transfer matrix method. Contour plots for f(θ,ω) as functions of both the excitation wavelength and the incidence angle for TM excitation are shown in fig. S2. Last, the normalized slowdown measure is calculated using the FIE and can be written as $\zeta \left(\theta ,\lambda \right)=f\left(\theta ,\lambda \right)\times \lambda^{2}$.

## Supplementary Materials

### This PDF file includes:

Sections S1 to S6 Figs. S1 to S6 Tables S1 to S3 References
