# Supplementary material for: Control of ultrafast hot electron dynamics in epsilon-near-zero conductive oxide thin films
Source: Sci Adv. 2025 May 21;11(21):eadu8850. doi: 10.1126/sciadv.adu8850 (PMC12094231; doi:10.1126/sciadv.adu8850)
Supplement: Supplementary file 1 — Sections S1 to S6 Figs. S1 to S6 Tables S1 to S3 References [file sciadv.adu8850_sm.pdf]

Science Advances
AAAS

Supplementary Materials for

Control of ultrafast hot electron dynamics in epsilon-near-zero conductive oxide thin films

Sudip Gurung *et al.*

Corresponding author: Ho Wai Howard Lee, howardhw.lee@uci.edu

Sci. Adv. **11**, eadu8850 (2025)
DOI: 10.1126/sciadv.adu8850

This PDF file includes:

Sections S1 to S6
Figs. S1 to S6
Tables S1 to S3
References

Supplementary Text

Section S1: Modeling the linear optical properties

The optical characteristics and thickness of the AZO nanolayer were assessed at angles of 55°, 60°, and 65° utilizing a (SE) spectroscopic ellipsometer (AngstromSun Technologies) with a spectral range from 400 to 1700 nm. The thickness of the AZO nanolayers and ITO was confirmed with a physical measurement with profilometer. The evaluation of surface roughness in the AZO nanolayers was performed using a Bruker-manufactured atomic force microscope. Wavelength dependence of the complex dielectric permittivity is modeled using the Drude model (*39*),

$$\varepsilon = \varepsilon' + i\varepsilon'' = \varepsilon_\infty - \frac{\omega_p^2}{\omega(\omega + i\gamma)} \tag{1}$$

$$\lambda_{\mathrm{ENZ}} = \frac{2\pi c}{\sqrt{\omega_{sp}^2 - \gamma^2}} \tag{2}$$

$$\varepsilon''_{\mathrm{ENZ}} = \frac{\varepsilon_\infty \gamma \lambda_{\mathrm{ENZ}}}{2\pi c} = \frac{\varepsilon_\infty \gamma}{\sqrt{\omega_{sp}^2 - \gamma^2}} \tag{3}$$

$$\omega_{\mathrm{ENZ}}^2 = \omega_{sp}^2 - \gamma^2 \tag{4}$$

$$\omega_{sp} = \frac{\omega_p}{\sqrt{\varepsilon_\infty}} \tag{5}$$

Where ε_∞ is the high-frequency response of the lattice, ω - angular frequency, c - speed of light, ω_p- plasma frequency, ω_{SP} -screened plasma frequency, γ - dephasing rate, λ_{ENZ} - ENZ wavelength and ε''_{ENZ} - imaginary permittivity at ENZ wavelength. The values of ε_∞, γ, and ω_p and thickness of the ENZ thin films are extracted from the SE fitting. A detailed study on the ALD AZO and optical characterization was reported in reference (*1*). The permittivity dispersion of the different ENZ samples is shown in fig. S1 and table S1.

Section S2: Field intensity enhancement inside the ENZ thin films

As a result of ENZ mode excitation, a relatively larger FIE is observed near the ENZ wavelength and close to the critical angle, i.e., 43.8° (*31*). The $f(\theta, \omega)$ for the ITO sample is slightly larger than that of the AZO sample because of the low loss of the ITO sample. It is important to note that the maximum field intensity enhancement (FIE) at normal incidence is consistently blue shifted relative to the ENZ wavelength. This shift occurs primarily due to the mode's hybridization as the result of the film's thickness and the excitation geometry. In particular, the maximum FIE wavelength is 1180 nm for ITO and 1370 nm for AZO. This enhanced $f(\theta, \omega)$ along with the incident light frequency/wavelength, i.e., normalized slowdown measure $\zeta(\theta, \lambda) = f(\theta, \lambda) \times \lambda^2$, is a strong tool for predicting the dependence of hot electron relaxation time on the ENZ mode excitation. This normalized slowdown measure follows from Equation (1) of the main text.

Section S3: The pulse duration of the laser pulses

We used a tunable Ti:Sapphire laser with ~ 60-fs FWHM 800-nm pulse measured using FROG at 1 kHz repetition rate. The 800-nm fundamental pulse drives an optical parametric amplification (OPA) to output a tunable range of 1150-1600 nm for our experiments. The infrared pulse duration was estimated with the inverse Fourier transform of the measured spectrum at each wavelength. Although these values give the minimum achievable pulse width at a particular wavelength, the pulse width at the sample also depends on the group velocity dispersion of media along the optical path, so the exact pulse width of the

laser pulses is typically slightly longer. Fig. S3 shows our estimation of the pulse duration dependence on the central laser wavelength based on our experimental measurements. The Ti:Sapphire laser specification was rated to have pulse width of around 60 fs at 800 nm (seed laser central wavelength), and our measured transform-limited pulse duration were also confirmed to have a pulse duration of around 60 fs in the near infrared wavelength. These values are used in the two-temperature model to fit normalized transmittance vs. delay data from the experiments.

Section S4: The hydrodynamic model (HDM)

A different approach to understand free electron ultrafast dynamics is the classical kinematic approach, where plasma is regarded as a dilute gas of N electrons enclosed in a box of volume V. The electron temperature T_e is assumed to be high enough, and their density is low enough, that they are localized in wave packets with the de-Broglie wavelength much smaller than their average separation. These criteria are fully met in typical optical experiments. Thus, electrons are regarded as distinguishable classical particles with charge $-e$, v is velocity vector, and electron mass (m). The electron dynamics-governed energy flow can be modeled by the following hydrodynamic equations [9],

$$\partial_t n + \nabla \cdot (nv) = 0, \tag{6}$$

$$\partial_t v + (v \cdot \nabla)v + \frac{3k_B}{mn}\nabla(nT_e) = \frac{1}{m}F_{\text{eff}} - \gamma \mathcal{F}_\gamma(v, T_e)v, \tag{7}$$

$$\partial_t T_e + \frac{2}{3}T_e \nabla \cdot v + v \cdot \nabla T_e = Q(v, T_e), \tag{8}$$

where, $F_{\text{eff}} = -eE - ev \times B$ is the effective external force, n is the carrier density, γ is the linear dephasing rate, k_B is the Boltzmann constant, $\mathcal{F}_\gamma(v, T_e)$ and $Q(v, T_e)$ are nonlinear current damping and hot electron heating dynamics terms explicitly given by

$$\mathcal{F}_\gamma(v, T_e) = \frac{3v_{T_0}^3}{2v^2 v_T}\left[\mathcal{G}(v/v_T) - e^{-\frac{v^2}{v_T^2}}\right] \tag{9}$$

$$Q(v, T_e) = \gamma_{\text{th}} \frac{M v_{T_0}^3}{2k_B v_T}\left[\mathcal{G}(v/v_T) - \frac{T_e v_{T_0}^2}{T_0 v_T^2} e^{-\frac{v^2}{v_T^2}}\right] \tag{10}$$

Eqs. (9) and (10) were analytically derived by transforming the Boltzmann equation into the nonlinear Fokker-Planck equation through the weak-coupling approach (*29*), obtaining

$$\mathcal{G}(v/v_T) = \frac{\sqrt{\pi}\, v_T \operatorname{erf}(v/v_T)}{2v} \tag{11}$$

$$v_T = \sqrt{2\, k_B (T_0/M + T_e/m)} \tag{12}$$

$$v_{T_0} = v_T(T_0) \tag{13}$$

$$\gamma_{th} = 2m\gamma/(m + M) \tag{14}$$

Here γ is the dephasing rate, γ_{th} is the hot electron relaxation rate describing relaxation towards equilibrium via electron-phonon scattering at low intensity, $\mathrm{v}_{\mathrm{T}_0}$ is the thermal velocity of electrons at room temperature T_0, and M is the mass of ions. In the presence of an external monochromatic field $E(t) = Re\left[E_0 e^{-i\omega t}\right]$, where $E_0 = E_0 \hat{e}_x$, the solution of the hydrodynamic equations can be obtained

perturbatively by setting $v = Re\left[(v_1 + v_3|E_0|^2)e^{-i\omega t}\right] + \mathcal{O}(|E_0|^4)$ and $T_e = T_0 + T_1|E_0|^2 + \mathcal{O}(|E_0|^4)$ and by neglecting nonlocal and higher-order terms,

$$\frac{\partial v_x}{\partial t} + \frac{e}{m}\langle E_{\mathrm{IN}} \cdot \hat{e}_x \rangle_z = \gamma \frac{3\sigma^3}{4\pi}\left\{-{v_x}^{-3}\mathrm{erf}\left[\frac{v_x}{v_T}\right] + \frac{2}{v_T\sqrt{\pi}}{v_x}^{-2}e^{-\frac{{v_x}^2}{{v_T}^2}}\right\}v_x \tag{15}$$

$$\frac{\partial T_e}{\partial t} = \gamma T_0 \frac{\sigma}{v_T}\left\{\frac{v_T}{v_x}\mathrm{erf}\left[\frac{v_x}{v_T}\right] - \frac{4\gamma T_e}{\sqrt{\pi}[\gamma_{\mathrm{th}}T_0 + (2\gamma - \gamma_{\mathrm{th}})T_e]}e^{-\frac{{v_x}^2}{{v_T}^2}}\right\} \tag{16}$$

where $v_T(x,t) = \sqrt{\frac{2k_B}{(2\gamma-\gamma_{\mathrm{th}})m}[\gamma_{\mathrm{th}}T_0 + (2\gamma - \gamma_{\mathrm{th}})T_e(x,t)]}$ and $\sigma = \sqrt{\frac{4\pi\gamma k_B T_0}{m(2\gamma-\gamma_{\mathrm{th}})}}$.

To understand the power dependence of the relaxation rate, we consider a TM-impinging electromagnetic pulse in the slowly varying envelope approximation (SVEA). In turn, we linearize the hydrodynamical equation for the average electron velocity driven by the spatial average of the electric field above, obtaining

$$\frac{\partial v_x}{\partial t} + \frac{e}{m}\langle E_{\mathrm{IN}} \cdot \hat{e}_x \rangle_z = -\gamma v_x \tag{17}$$

where $v_x = Re\left[v_0 e^{-i\omega t}\right]$and $\langle E_{\mathrm{IN}} \cdot \hat{e}_x \rangle_z = Re\left[E_{\mathrm{AV}}e^{-i\omega t}\right]$, leading to $v_0 = \frac{-eE_{\mathrm{AV}}}{m(\gamma - i\omega)}$. Also, $\langle v_x^2 \rangle = \frac{1}{2}|v_0|^2 = \frac{1}{2}\frac{e^2}{m^2(\omega^2+\gamma^2)}|E_{\mathrm{AV}}|^2 = \frac{I_0}{I_s}$, where $I_0(t) = \frac{1}{2}\varepsilon_0 c|E_0(t)|^2$ is the instantaneous intensity of the impinging field, $I_s = \frac{\varepsilon_0 c m^2(\omega^2+\gamma^2)}{e^2 f(\theta,\omega)}$ is the intensity per squared velocity, and $f(\theta,\omega)$ is the electric field enhancement factor.

The time evolution of the electron temperature can be plotted using Eq. (16). Fig. S4 illustrates the normalized change in electron temperature using the hydrodynamic and two-temperature models, which is then compared to the normalized experimental transmission.

From $v_T = \sqrt{\frac{2k_B}{(2\gamma-\gamma_{\mathrm{th}})m}[\gamma_{\mathrm{th}}T_0 + (2\gamma - \gamma_{\mathrm{th}})T_e]}$, we have $\frac{2k_B}{(2\gamma-\gamma_{\mathrm{th}})m} = \frac{v_T^2}{\gamma_{\mathrm{th}}(T_0 - T) + 2\gamma T}$, and using $\mathrm{erf}\left[\frac{v}{v_T}\right] \approx \frac{2}{\sqrt{\pi}}\left[\frac{v}{v_T} - \frac{v^3}{3\mathrm{v}_T^3} + \frac{v^5}{10\mathrm{v}_T^5} + O(\frac{v^7}{v_T^7})\right]$, we obtain:

$$\gamma_r = \gamma_{\mathrm{th}}\frac{\sqrt{\pi}v_T}{2v}\mathrm{erf}\left[\frac{v}{v_T}\right] = \gamma_{\mathrm{th}}v_T\sqrt{\frac{\pi I_s}{4I_0}}\mathrm{erf}\left[\frac{1}{v_T}\sqrt{\frac{I_0}{I_s}}\right] \approx \gamma_{\mathrm{th}}\left[1 - \frac{1}{3\mathrm{v}_T^2}\sqrt{\frac{I_0^2}{I_s^2}}\right]$$
$$= \gamma_{\mathrm{th}} - \frac{e^2\gamma_{\mathrm{th}}(2\gamma - \gamma_{\mathrm{th}})f(\theta,\omega)}{12\varepsilon_0 c\gamma m k_B T_0(\gamma^2 + \omega^2)}I_0 \tag{18}$$

Thus, using the first-order approximation of the series expansion of $1/\gamma_r$, the intensity-dependent relaxation time is calculated as

$$\tau_r = \gamma_{\mathrm{th}}^{-1} + \frac{\gamma_{\mathrm{th}}^{-1}e^2(2\gamma - \gamma_{\mathrm{th}})f(\theta,\omega)}{12\varepsilon_0 c\gamma m k_B T_0(\gamma^2 + \omega^2)}I_0. \tag{19}$$

Eq. (19) shows that the nonlinear relaxation time τ_r depending on the intensity I_0, the angle of incidence and wavelength through the field intensity enhancement factor $f(\theta,\omega)$. It is worth noting that the linear

relaxation time τ_{th} ($= {\gamma_{\text{th}}}^{-1}$) is estimated from the intensity-dependent relaxation time experiment [see main text Fig. 3(b)] by extrapolating the relaxation time at very low intensity. The linear relaxation times (τ_{th}) for AZO and ITO are 18 fs and 42 fs, respectively (see Table S1).

The mechanism of enhanced hot electron relaxation time can be understood as a reduction of Coulomb interaction between electrons and ions for highly energetic electrons, resulting in less deflection of the highly energetic electrons. Lowering the deflection of highly energetic electrons reduces the probability of electron-electron and electron-phonon scattering, resulting in an increase in hot electron relaxation time (τ_r), as illustrated in (see main text Fig. 1(b)). The lower the deflection for highly energetic electrons, the less energy is transferred from the optical pulse to the electron plasma.

Section S5: Two-temperature model (TTM)

The two-temperature model (TTM) describes the dynamics of electrons and phonons as thermalization involving two coupled thermal reservoirs. This concept provides the most straightforward and intuitive description of the thermalization of electronic and vibrational degrees of freedom in out-of-equilibrium systems, and it has found widespread application in the description of ultrafast processes in solids. The underlying ideas of the TTM are best demonstrated by considering the thermalization dynamics of the two systems, whose interactions are governed by the laws of thermodynamics. In the presence of the interaction between the systems, the energy transfer depends on the difference in temperature between them.

For intraband transition, the energy of the light pulse absorbed by the material is transferred to non-thermalized electrons (known as "hot electrons") by promoting free electrons lying below the Fermi level to the unoccupied levels above the Fermi level (*20*). These electrons absorb photons without a change in electron temperature (non-thermalized electrons or hot electron distribution process). The non-thermalized electrons then transfer energy to surrounding electrons through electron-electron scattering, raising the temperature of the surrounding electrons (thermalized electron distribution process). Finally, the thermalized electrons undergo electron-phonon collisions to regain thermal equilibrium. As a result, the above energy-flowing processes in TTM can be described using the following three coupled differential equations:

$$\frac{\partial N(t)}{\partial t} = -(\gamma_e + \gamma_p)N + P_A(t) \tag{20}$$

$$C_e \frac{\partial T_e(t)}{\partial t} = g_{e-p}(T_l - T_e) + \gamma_e N(t) \tag{21}$$

$$C_l \frac{\partial T_l(t)}{\partial t} = -g_{e-p}(T_l - T_e) + \gamma_p N(t) \tag{22}$$

where C_l and C_e are the heat capacities of lattice and electron subsystems, γ_e and γ_e are electron-electron and electron-phonon thermalization rates, N and P_A are the total number of carriers and the number of absorbed optical photons. Certain assumptions were applied to facilitate the investigation of the above differential equations,

a) In intra-band pumping ($\hbar\omega < E_{bg}$), there is no change in the total carrier population,
b) Change in the specific heat capacity of the lattice is larger than electrons because ($\mathrm{C_l} \gg \mathrm{C_e}$),
c) Change in the lattice temperature is negligible.

Note that assumption (b) is used after observing the pump-probe experiment data, where the transmission change falls back to its original value after the optical pulse passes through (see fig. S4(a)). Similar

assumptions using TTM have been made to study the ultrafast nonlinear nature of the TCO (*20*). Change in normalized electron temperature can be written as (*29*)

$$\frac{\Delta T}{\Delta T_{\max}} = \int_{-\infty}^{+\infty} h_T(t')\, e^{-2\left(\frac{t'}{t_{\text{pump}}}\right)^2} dt' \quad (23)$$

$$h_T(t) = \Theta(t)(\tau_t - \tau_r)^{-1}\left(e^{-t/\tau_t} - e^{-t/\tau_r}\right) \quad (24)$$

$$\tau_r = \frac{C_e C_l}{g_{e-p}(C_e + C_l)} \quad (25)$$

$h_T(t)$ is the temporal response of the material, $\Theta(t)$ is the Heaviside step function, and $\tau_t = \frac{1}{\gamma_e+\gamma_p}$ is the time at which the nonthermal electrons release energy to thermalized ones. Ignoring $\gamma_p \ll \gamma_e$, (*39*) we assume $\tau_t \approx \frac{1}{\gamma_e}$, where $\gamma_e \sim \gamma$ is the damping rate, t_{pump} is the full temporal width at half maxima of the pump beam, g_{e-p} is the electron-phonon coupling coefficient, and τ_r is the relaxation time of the thermalized electrons with the lattice. Here τ_r is not independent of electron temperature since C_e and g_{e-p} are not considered constants. However, the assumption that the time required for nonthermal electrons to release energy to thermal electrons is considered constant, and this assumption leads to a difference in the relaxation time between HDM and TTM.

Note that the normalized rate of change in transmission from the pump-probe experiment is because of the change in electron temperature. Therefore, the normalized transmission and normalized change in electron temperature must have a linear relationship. We plotted normalized transmittance against normalized change in electron temperature as shown in fig. S4 (b) and (c), where (b) represents the rising part, whereas (c) represents the falling part of the temperature/transmission. A black dashed straight line represents a one-to-one linear relationship. The one-to-one linear relationship is evident in a close correspondence between the normalized transmission and normalized change in electron temperature with the straight line. The normalized change in electron temperature was fitted using a linear equation (y = mx+c), where the slope "m" was found to be ≈ 1 (see fig. S4 (b) and (c)). The deviation of normalized change in electron temperature from the straight line can be attributed to the fluctuation of the measured normalized transmittance, i.e., experimental error in the measurement of the transmitted probe beam. Thus, the normalized transmission experimental data can be fitted using Eq. (23). The value of τ_r is derived by fitting experimental pump-probe data via robust nonlinear regression using Eq. (24). The theoretical prediction of the relaxation time using the TTM, however, becomes very challenging (*21, 40*) because, according to the two-temperature model, electron-phonon scattering is related to the electron (C_e), lattice (C_l) heat capacity, and coupling constant (g_{e-p}) by ($\tau_r = \frac{C_e C_l}{g_{e-p}(C_e+C_l)}$), and all of these quantities have a complex relationship with high electron and lattice temperatures. The theoretical prediction of τ_r dependence on the intensity of impinging pulse becomes complicated because both the heat capacities and the coupling coefficient are intensity dependent.

Section S6: Dependence of relaxation time on wavelength and incident angle

This section shows the dependence of the relaxation time at different angles of incidence and wavelength. Fig. S5 shows the dependence of relaxation time on the angle of incidence. Contour maps of the normalized experimental transmission of probe ($\Delta T/\Delta T_{max}$) (fig. S5(a,d)) and normalized electron temperature ($\Delta T_e/\Delta T_e^{max}$) (fig. S5 (b,e)) with varying delay (fs) between pump and probe at different angles of incidence for 100 GW/cm^2 peak pump intensity in AZO and ITO samples are shown. The pump and probe wavelength are at the ENZ wavelength of AZO and ITO, respectively. We observed

that the recovery time for the normalized transmittance followed the field intensity enhancement ($f(\theta,\lambda)$), which is consistent with the simulated normalized electron temperature versus delay with different angles of incidence using the HDM. Angular dependence of the relaxation time is shown in fig. S5 (f). As predicted by the hydrodynamic model, a prominent angular dependence of the relaxation time with the $f(\theta,\omega)$ is seen in both AZO and ITO.

Similarly, fig. S6 (a,d) and (b,e) shows respectively the contour maps of the normalized experimental transmission of the probe ($\Delta T/\Delta T_{max}$) and normalized electron temperature ($\Delta T_e/\Delta T_e^{max}$) vs. delay (fs) between pump and probe at different wavelengths for peak pump intensity of 100 GW/cm^2 in AZO and ITO. The pump beam is at a normal angle of incidence. As predicted in the main text, the relaxation time strongly depends on the Figure of merit ($\zeta(\theta,\lambda) = f(\theta,\lambda) \cdot \lambda^2$). We observed that the recovery time for the normalized transmittance, which corresponds to normalized electron temperature, followed the $\zeta(\theta,\lambda)$, consistent with the HDM.

Fig. S1: Dispersion of the ENZ samples, (a) AZO, and (b) ITO

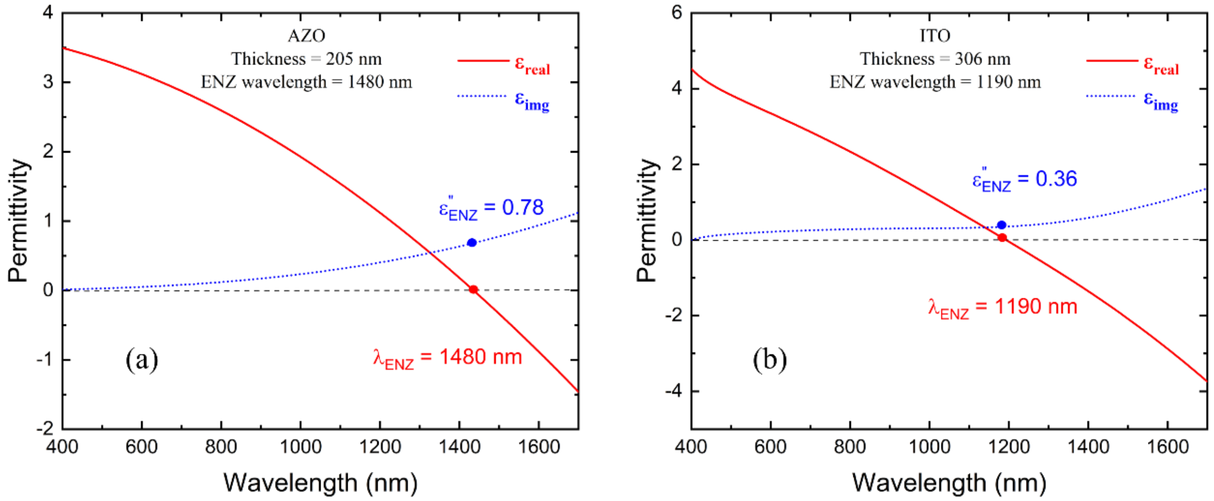

Figure S1: Dispersion of the ENZ samples, (a) AZO, and (b) ITO

Fig. S2: Field intensity enhancement ($f(\theta,\omega)$ or FIE) for TM polarization at different excitation wavelengths and incidence angles for ITO and AZO samples

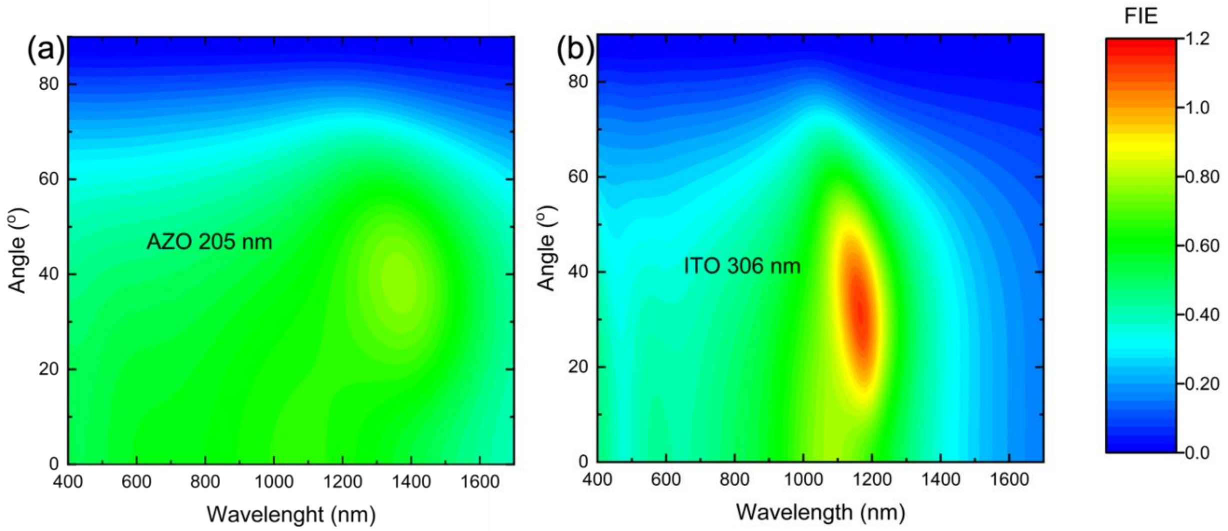

Figure S2: Field intensity enhancement ($f(\theta,\omega)$ or FIE) for TM polarization at different excitation wavelengths and incidence angles for (a) AZO (205 nm) and (b) ITO (306 nm).

Fig. S3: Pulse duration dependence on the central wavelength of the laser pulses

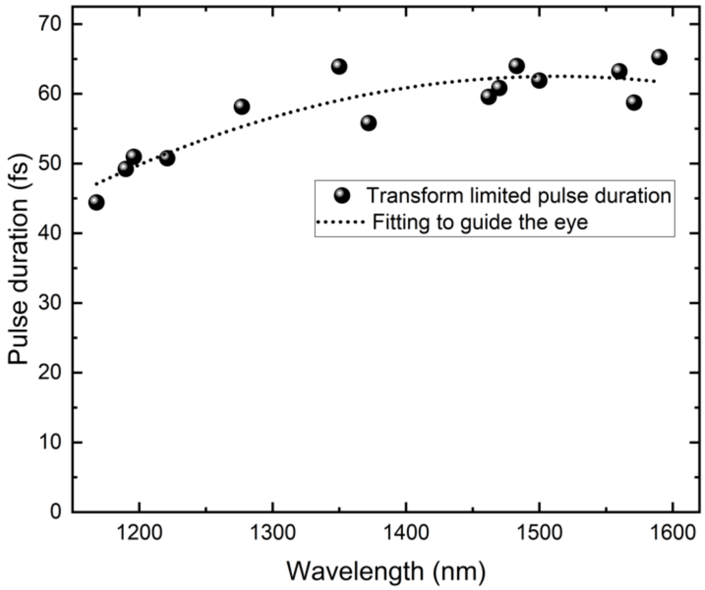

Figure S3: Pulse duration dependence on the central wavelength of the laser pulses.

Fig. S4: Normalized transmission vs. delay predicted by the hydrodynamic model and fitted using the two-temperature model

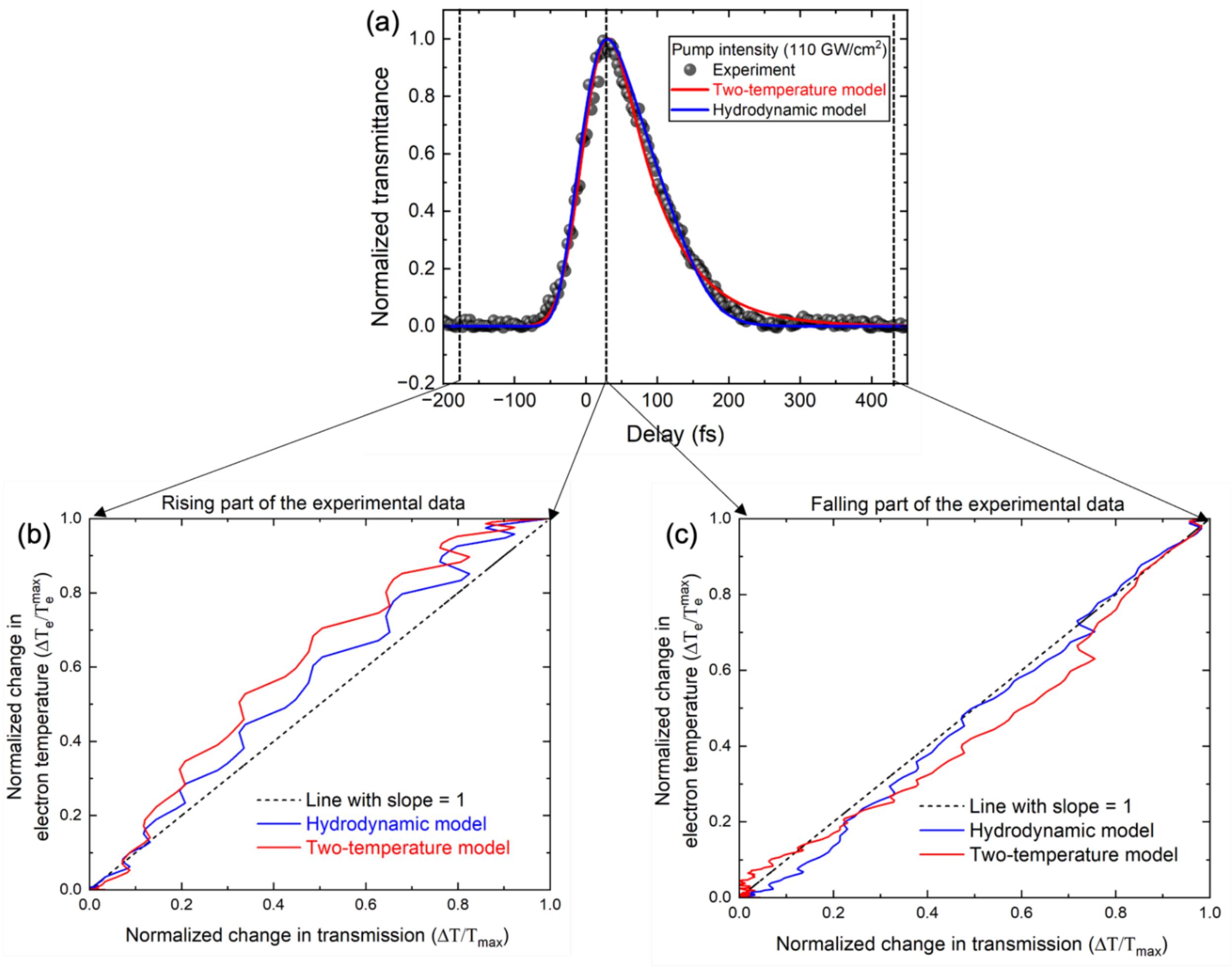

Figure S4: (a) Normalized transmission vs. delay at 110 GW/cm2 predicted by the hydrodynamic model and fitted using the two-temperature model for 205 nm AZO at ENZ wavelength. Note that the two-temperature model data is derived by fitting the experimental data, whereas the hydrodynamic model data is provided from the theoretical calculation. A linear relationship between the normalized transmission and normalized temperature was observed (b) for the rising part and (c) for the falling part. This linear relationship allows us to compare the normalized transmission with the normalized change in electron temperature.

Fig. S5: Contour map of the normalized experimental transmission of the probe and normalized electron temperature vs. delay (fs) between pump and probe at different angles of incidence for ITO and AZO samples

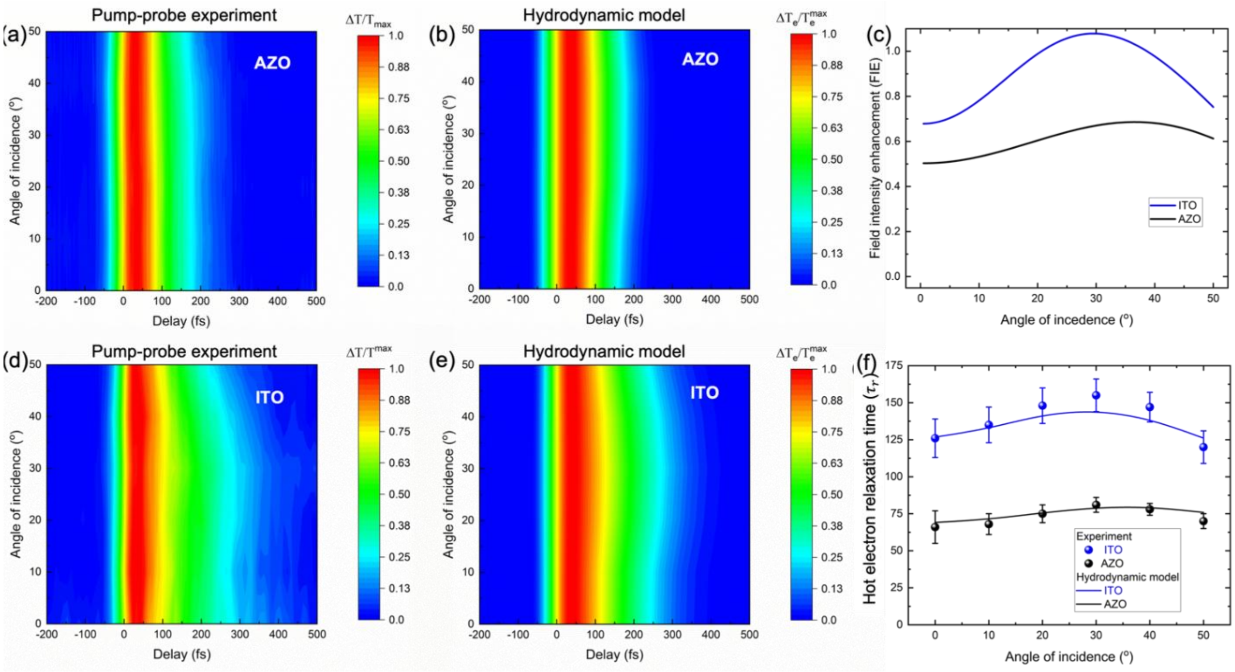

Figure S5: Contour map of the normalized experimental transmission of the probe ($\Delta T/\Delta T_{max}$) (a,d) and normalized electron temperature ($\Delta T_e/\Delta T_e^{max}$) (b,e) vs. delay (fs) between pump and probe at different angles of incidence for 100 GW/cm^2 peak pump intensity in AZO and ITO samples. The pump and probe wavelength are fixed to the ENZ wavelengths of AZO and ITO film. (c) Field intensity enhancement vs. angle of incidence for AZO and ITO at their respective ENZ wavelength (1480 nm and 1190 nm) (f) Comparison of the magnitude of hot electron relaxation time at different angles of incidence for AZO and ITO samples. Circles are derived by fitting the experimental data using the two-temperature model, and lines are the hydrodynamic model predictions.

Fig. S6: Contour map of the normalized experimental transmission of the probe and normalized electron temperature vs. delay (fs) between pump and probe at different wavelengths for ITO and AZO samples

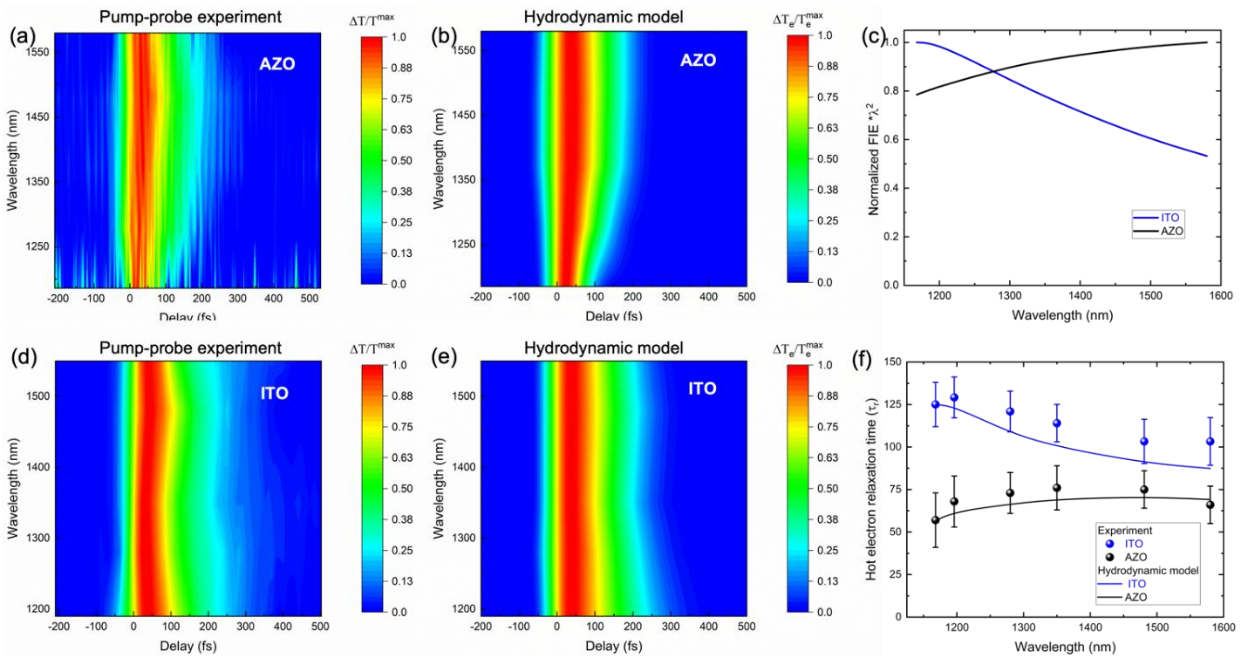

Figure S6: Contour map of the normalized experimental transmission of probe ($\Delta T/\Delta T_{max}$) **(a,d)** and normalized electron temperature ($\Delta T_e/\Delta T_e^{max}$) **(b,e)** vs. delay (fs) between pump and probe at different wavelengths for 100 GW/cm^2 peak pump intensity in AZO and ITO samples. The pump beam is at a normal angle of incidence. **(c)** Field intensity enhancement times the square of wavelength ($f(\theta, \lambda) \cdot \lambda^2$) at a different wavelength for AZO and ITO **(f)** Comparison of the magnitude of hot electron relaxation time at different wavelengths for AZO and ITO samples. Circles are derived by fitting the experimental data using the two-temperature model, and lines are the hydrodynamic model predictions.

Table S1: ENZ parameters and optical properties for 2 different ENZ samples used in the pump-probe measurements

Parameters	*AZO (ALD)*	*ITO (Sputtered)*
Thickness	205 nm	306 nm
$\boldsymbol{\lambda}_{\mathbf{ENZ}}$ (ENZ wavelength)	1480 nm	1190 nm
$\boldsymbol{\lambda}_{\mathbf{P}}$ (Plasma wavelength)	788 nm	621 nm
$\boldsymbol{\varepsilon}''_{\mathbf{ENZ}}$ (Imaginary permittivity at λ_{ENZ})	0.78	0.36
$\mathbf{T}_{\mathbf{eq}}$	300 K	300 K
$\mathbf{1/\boldsymbol{\gamma}}$	4.34 fs	6.36 fs
$\boldsymbol{\tau}_{\mathbf{th}} \sim \mathbf{1}/\boldsymbol{\gamma}_{\mathbf{th}}$	21 fs	42 fs
$\mathbf{m}_{\mathbf{eff}}$ (effective mass)	0.36 $\mathrm{m_e}$	0.38 $\mathrm{m_e}$
$\boldsymbol{\theta}$ (angle between pump and probe)	10°	10°

Table S1: ENZ parameters and optical properties for 2 different ENZ samples used in the pump-probe measurements

Table S2: Summary of Hot Electron Relaxation Times in ENZ Materials

Material	*ENZ wavelength (nm)*	*Pump wavelength (nm)*	*Probe wavelength (nm)*	*Relaxation time (fs)*	*Intensity (GW/cm²)*	*Approach*	*Reference*
AZO	1480	1480	1480	31.4 - 89.6	20-200	TTM + HDM	**This work**
ITO	1190	1190	1190	62.6 - 169.5	20-200	TTM + HDM	**This work**
ITO	1160	1028	1080	160 - 200	12-37	TTM	[24]
ITO	1212	1250	1000-1650	30	50	Extended Drude model	[19]
ITO	1200	790	1200	170	35	TTM	[20]
ITO	1240	1240	1240	360	NA	TTM	[12]
Multi-layer Au/TiO_2	605	470	590	3300	13	TTM	[41]
AZO	1860	1500-2000	550-950	350	NA	Exponential fitting	[42]

Table S2: Summary of Hot Electron Relaxation Times in ENZ Materials

Table S3: Experiment specification

Equipment	*Vendor + model/part number*	*Operating wavelength*
**Femtosecond laser**	Coherent Ti:Sapp Laser with OPA	1100 – 1600 nm
**Linear polarizer**	Thorlabs LPNIR100-MP2	650 – 2000 nm
**90:10 Beam splitter**	Thorlabs BSN12R	1200 – 1600 nm
**Half-wave plate**	Thorlabs AHWP10M-1600	1100 – 2000 nm
**Probe focusing lens**	Thorlabs AC254-150-C-ML (f =150 mm)	1050 – 1700 nm
**Pump focusing lens**	Thorlabs AC254-200-C-ML (f =200 mm)	1050 – 1700 nm
**Power sensor**	Newport 918D-ST-IR	780 – 1800 nm
**Delay stage**	Thorlabs ODL300	
**Power meter**	Newport 2936-R	

Table S3: Experiments optical components and light source

REFERENCES AND NOTES

1. S. Gurung, A. Anopchenko, S. Bej, J. Joyner, J. Myers, J. Frantz, H. Lee, Atomic layer engineering of epsilon-near-zero ultrathin films with controllable field enhancement. *Adv. Mater. Interfaces* **7**, 2000844 (2020).

2. A. Anopchenko, S. Gurung, L. Tao, C. Arndt, H. Lee, Atomic layer deposition of ultra-thin and smooth Al-doped ZnO for zero-index photonics. *Mater. Res. Express* **5**, 014012 (2018).

3. G. Naik, V. Shalaev, A. Boltasseva, Alternative plasmonic materials: Beyond gold and silver. *Adv. Mater.* **25**, 3264–3294 (2013).

4. S. Vassant, J. Hugonin, F. Marquier, J. Greffet, Berreman mode and epsilon near zero mode. *Opt. Express* **20**, 23971–23977 (2012).

5. S. Campione, I. Brener, F. Marquier, Theory of epsilon-near-zero modes in ultrathin films. *Phys. Rev. B* **91**, 121408 (2015).

6. S. Campione, I. Kim, D. D. Ceglia, G. A. Keeler, A. T. S. Luk, Experimental verification of epsilon-near-zero plasmon polariton modes in degenerately doped semiconductor nanolayers. *Opt. Express* **24**, 18782–18789 (2016).

7. S. Campione, D. de Ceglia, M. Vincenti, M. Scalora, F. Capolino, Electric field enhancement in ε-near-zero slabs under TM-polarized oblique incidence. *Phys. Rev. B* **87**, 035120 (2013).

8. W. Tian, F. Liang, S. Chi, C. Li, H. Yu, H. Zhang, H. Zhang, Highly efficient super-continuum generation on an epsilon-near-zero surface. *ACS Omega* **5**, 2458–2464 (2020).

9. Y. Yang, J. Lu, A. Manjavacas, T. Luk, H. Liu, K. Kelley, J. Maria, E. Runnerstrom, M. Sinclair, S. Ghimire, I. Brener, High-harmonic generation from an epsilon-near-zero material. *Nat. Phys.* **15**, 1022–1026 (2019).

10. W. Jaffray, F. Belli, S. Stengel, M. Vincenti, M. Scalora, M. Clerici, V. Shalaev, A. Boltasseva, M. Ferrera, High-order nonlinear frequency conversion in transparent conducting oxide thin films. *Adv. Opt. Mater.* **12**, 2401249 (2024).

11. W. Tian, F. Liang, D. Lu, H. Yu, H. Zhang, Highly efficient ultraviolet high-harmonic generation from epsilon-near-zero indium tin oxide films. *Photonics Res.* **9**, 317–323 (2021).

12. M. Alam, I. De Leon, R. Boyd, Large optical nonlinearity of indium tin oxide in its epsilon-near-zero region. *Science* **352**, 795–797 (2016).

13. N. Kinsey, C. DeVault, J. Kim, M. Ferrera, V. Shalaev, A. Boltasseva, Epsilon-near-zero Al-doped ZnO for ultrafast switching at telecom wavelengths. *Optica* **2**, 616–622 (2015).

14. S. Saha, A. Dutta, C. DeVault, B. Diroll, R. Schaller, Z. Kudyshev, X. Xu, A. Kildishev, V. Shalaev, A. Boltasseva, Extraordinarily large permittivity modulation in zinc oxide for dynamic nanophotonics. *Mater. Today* **43**, 27–36 (2021).

15. L. Caspani, R. Kaipurath, M. Clerici, M. Ferrera, T. Roger, J. Kim, N. Kinsey, M. Pietrzyk, A. Di Falco, V. Shalaev, A. Boltasseva, D. Faccio, Enhanced nonlinear refractive index in ε-near-zero materials. *Phys. Rev. Lett.* **116**, 233901 (2016).

16. O. Reshef, E. Giese, M. Alam, I. De Leon, J. Upham, R. Boyd, Beyond the perturbative description of the nonlinear optical response of low-index materials. *Opt. Lett.* **42**, 3225–3228 (2017).

17. E. Li, A. Wang, Femto-Joule all-optical switching using epsilon-near-zero high-mobility conductive oxide. *IEEE J. Sel. Top. Quantum Electron.* **27**, 1–9 (2021).

18. H. Wang, K. Du, C. Jiang, Z. Yang, L. Ren, W. Zhang, S. Chua, T. Mei, Extended drude model for intraband-transition-induced optical nonlinearity. *Phys. Rev. Appl.* **11**, 064062 (2019).

19. H. Wang, K. Du, R. Liu, X. Dai, W. Zhang, S. Chua, T. Mei, Role of hot electron scattering in epsilon-near-zero optical nonlinearity. *Nanophotonics* **9**, 4287–4293 (2020).

20. F. Zhang, C. Chen, K. Wang, H. Zhang, J. Chen, Rationale behind subpicosecond optical response of transparent conductive oxides in epsilon-near-zero region. *J. Appl. Phys.* **129**, 243101 (2021).

21. E. Carpene, Ultrafast laser irradiation of metals: Beyond the two-temperature model. *Phys. Rev. B* **74**, 024301 (2006).

22. N. Singh, Two-temperature model of nonequilibrium electron relaxation: A review. *Int. J. Mod. Phys. B* **24**, 1141–1158 (2010).

23. I. Un, S. Sarkar, Y. Sivan, Electronic-based model of the optical nonlinearity of low-electron-density drude materials. *Phys. Rev. Appl.* **19**, 044043 (2023).

24. A. Bykov, J. Deng, G. Li, A. Zayats, Time-dependent ultrafast quadratic nonlinearity in an epsilon-near-zero platform. *Nano Lett.* **24**, 3744–3749 (2024).

25. C. Voisin, N. Del Fatti, D. Christofilos, F. Vallée, Ultrafast electron dynamics and optical nonlinearities in metal nanoparticles. *J. Phys. Chem. B* **105**, 2264–2280 (2001).

26. Q. Guo, Y. Cui, Y. Yao, Y. Ye, Y. Yang, X. Liu, S. Zhang, X. Liu, J. Qiu, H. Hosono, A solution-processed ultrafast optical switch based on a nanostructured epsilon-near-zero medium. *Adv. Mater.* **29**, 1700754 (2017).

27. M. Silvestri, A. Sahoo, L. Assogna, P. Benassi, C. Ferrante, A. Ciattoni, A. Marini, Resonant third-harmonic generation driven by out-of-equilibrium electron dynamics in sodium-based near-zero index thin films. *Nanophotonics* **13**, 2003–2013 (2024).

28. J. Hajdu, R. Balescu, Equilibrium and nonequilibrium statistical mechanics John Wiley & Sons, Chichester, New York, Sydney, Toronto 1975, 742 Seiten, Preis: £ 16.20, Deutsche Bunsen-Gesellschaft für Physikalische Chemie 81, 240 (1977).

29. A. Marini, A. Ciattoni, C. Conti, Out-of-equilibrium electron dynamics of silver driven by ultrafast electromagnetic fields—A novel hydrodynamical approach. *Faraday Discuss.* **214**, 235–243 (2019).

30. M. Rosenbluth, S. Putvinski, Theory for avalanche of runaway electrons in tokamaks. *Nucl. Fusion* **37**, 1355–1362 (1997).

31. A. Anopchenko, S. Gurung, S. Bej, H. Lee, Field enhancement of epsilon-near-zero modes in realistic ultrathin absorbing films. *Nanophotonics* **12**, 2913–2920 (2023).

32. M. Scalora, M. Vincenti, D. de Ceglia, V. Roppo, M. Centini, N. Akozbek, M. Bloemer, Second- and third-harmonic generation in metal-based structures. *Phys. Rev. A* **82**, 043828 (2010).

33. F. De Luca, M. Ortolani, C. Ciracì, Free electron nonlinearities in heavily doped semiconductors plasmonics. *Phys. Rev. B* **103**, 115305 (2021).

34. B. Diroll, P. Guo, R. Chang, R. Schaller, Large transient optical modulation of epsilon-near-zero colloidal nanocrystals. *ACS Nano* **10**, 10099–10105 (2016).

35. S. Saha, B. Diroll, J. Shank, Z. Kudyshev, A. Dutta, S. Chowdhury, T. Luk, S. Campione, R. Schaller, V. Shalaev, A. Boltasseva, M. Wood, Broadband, high-speed, and large-amplitude dynamic optical switching with yttrium-doped cadmium oxide. *Adv. Funct. Mater.* **30**, 1980377 (2020).

36. Y. Wu, Y. Wang, M. Wang, H. Ma, H. Jiang, J. Yao, Y. Zhao, K. Yu, J. Shao, Tailoring epsilon-near-zero wavelength and nonlinear absorption properties of CdO thin films by Mo doping. *Appl. Phys. Lett.* **125**, 011104 (2024).

37. J. Huang, N. Dong, J. Wang, in *Two-Dimensional Materials for Nonlinear Optics: Fundamentals, Preparation Methods, and Applications,* Q. Wang, H.-L. Zhang, Eds. (Wiley-VCH, 2023), chap. 5, pp. 103–130.

38. D. Lei, D. Su, S. Maier, New insights into plasmonic hot-electron dynamics. *Light Sci. Appl.* **13**, 243 (2024).

39. V. Romanyuk, N. Dmitruk, V. Karpyna, G. Lashkarev, V. Popovych, M. Dranchuk, R. Pietruszka, M. Godlewski, G. Dovbeshko, I. Timofeeva, O. Kondratenko, M. Taborska, A. Ievtushenko, Optical and electrical properties of highly doped ZnO:Al films deposited by atomic layer deposition on si substrates in visible and near infrared region. *Acta Phys. Pol. A* **129**, A-36–A-40 (2016).

40. Z. Lin, L. Zhigilei, V. Celli, Electron-phonon coupling and electron heat capacity of metals under conditions of strong electron-phonon nonequilibrium. *Phys. Rev. B* **77**, 075133 (2008).

41. A. Rashed, B. Yildiz, S. Ayyagari, H. Caglayan, Hot electron dynamics in ultrafast multilayer epsilon-near-zero metamaterials. *Phys. Rev. B* **101**, 165301 (2020).

42. X. Tian, H. Luo, R. Wei, M. Liu, Z. Yang, Z. Luo, H. Zhu, J. Li, J. Qiu, Ultrafast and broadband optical nonlinearity in aluminum doped zinc oxide colloidal nanocrystals. *Nanoscale* **11**, 13988–13995 (2019).
